# Decreasing the Burden of West Nile Virus Disease: The Need for More Prevention Measures

**DOI:** 10.4269/ajtmh.26-0404

**Published:** 2026-09-08

**Authors:** Randall J. Nett, Aaron C. Brault, Casey Parker-Crockett, J. Erin Staples

**Affiliations:** Division of Vector-Borne Diseases, Centers for Disease Control and Prevention, Fort Collins, Colorado, USA

## Abstract

After the introduction of West Nile virus (WNV) in 1999, the virus became endemic in the United States, causing approximately 1,300 severe neurological disease cases and 130 deaths annually. WNV is also responsible for a substantial disease burden in Canada, Europe, and countries around the Mediterranean Sea. WNV prevention currently relies on personal protective behaviors (PPBs) and mosquito control, but these measures have been inadequate to substantially reduce WNV disease cases or deaths. Additional measures are also necessary to diagnose, treat, prevent, and control WNV disease. Human WNV vaccine, anti-WNV monoclonal antibody used as preexposure prophylaxis in patients at higher risk for severe WNV disease, and more rapidly detecting WNV infection, among other measures, will be critical to help combat WNV disease morbidity and mortality. Partners in government, academia, and private industry should collaborate to ensure optimal use and availability of current and additional lifesaving measures.

## INTRODUCTION

West Nile virus (WNV), transmitted by *Culex* spp. mosquitoes and maintained in a mosquito–bird–mosquito amplification cycle, emerged in the United States in 1999 and causes substantial morbidity and mortality each year.^1^^,^^2^ During the period 1999–2024, 61,009 WNV disease cases, including 31,818 severe West Nile neuroinvasive disease cases and 3,143 deaths, were reported in the United States.^3^ Public health has relied on use of personal protective behaviors (PPBs) and mosquito control to prevent WNV disease.^1^ Despite these efforts, WNV-related morbidity and mortality is not decreasing and WNV remains a large public health concern.

Although the PPBs and mosquito control efforts have likely prevented some WNV disease, they are unlikely to be successful alone at substantially reducing WNV disease burden to much lower levels. This limited effectiveness has also been observed with Japanese encephalitis virus, a *Culex*-borne virus with a bird reservoir, where mosquito control and health education alone did not reduce Japanese encephalitis incidence in Thailand. Incidence declined only after vaccination was introduced in high-risk areas alongside these other measures.^4^

Recognizing the need to improve WNV disease diagnosis, treatment, prevention, and control, the US Health and Human Services National Public Health Strategy to Prevent and Control Vector-Borne Diseases in People established a goal to reduce the annual number of WNV neuroinvasive disease cases to below 500 by 2035.^5^ To meet this goal, we need to improve the use of existing measures and develop additional measures for WNV prevention and control. This perspective reviews WNV epidemiology, describes current WNV prevention and control measures, and highlights some additional measures needed to address the goal of lowering the burden of WNV-related morbidity and mortality.

### WNV epidemiology.

**Figure 1.**
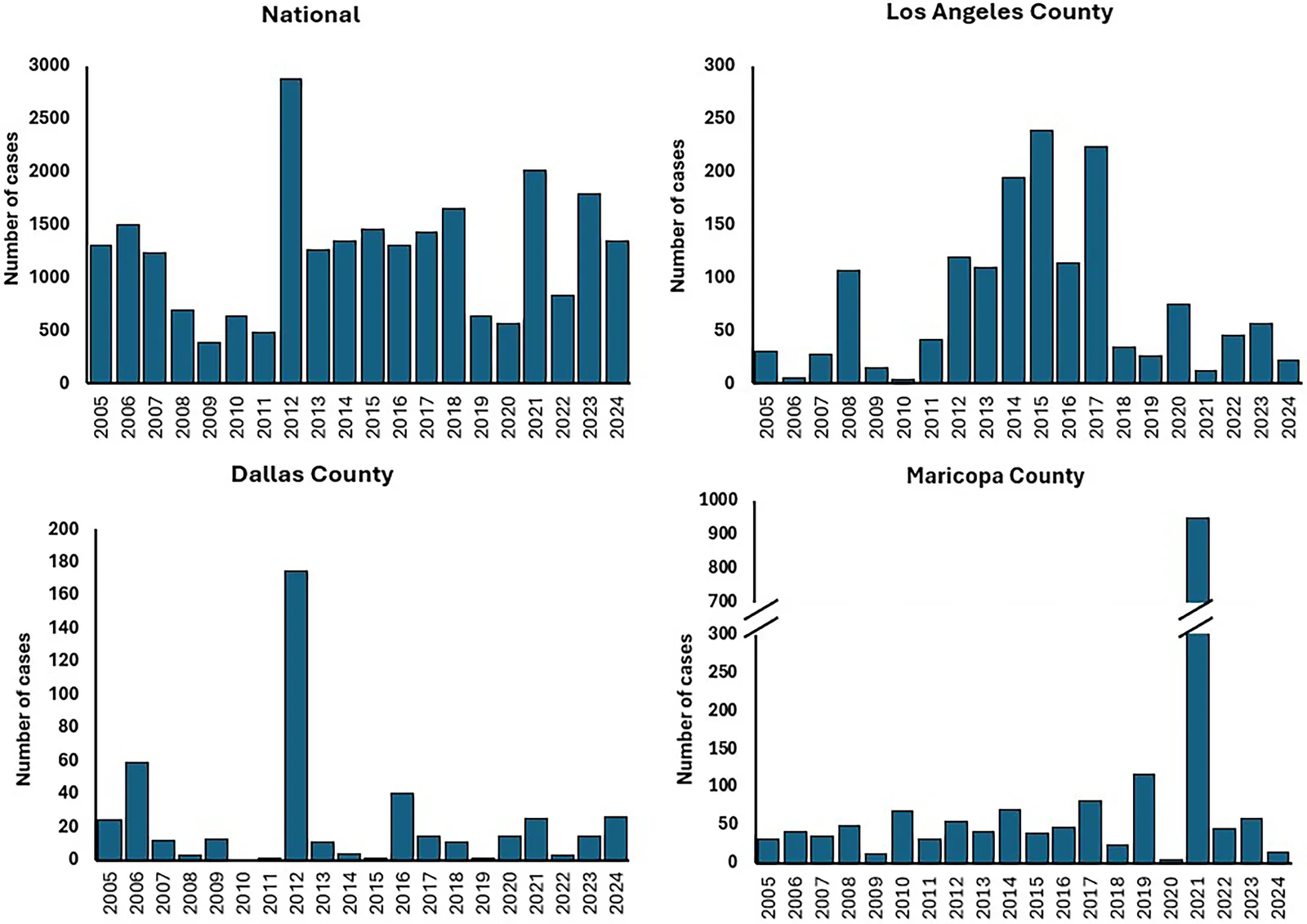
West Nile virus neuroinvasive disease cases by year nationally and in select U.S. metropolitan counties, 2005–2024.

**Figure 2.**
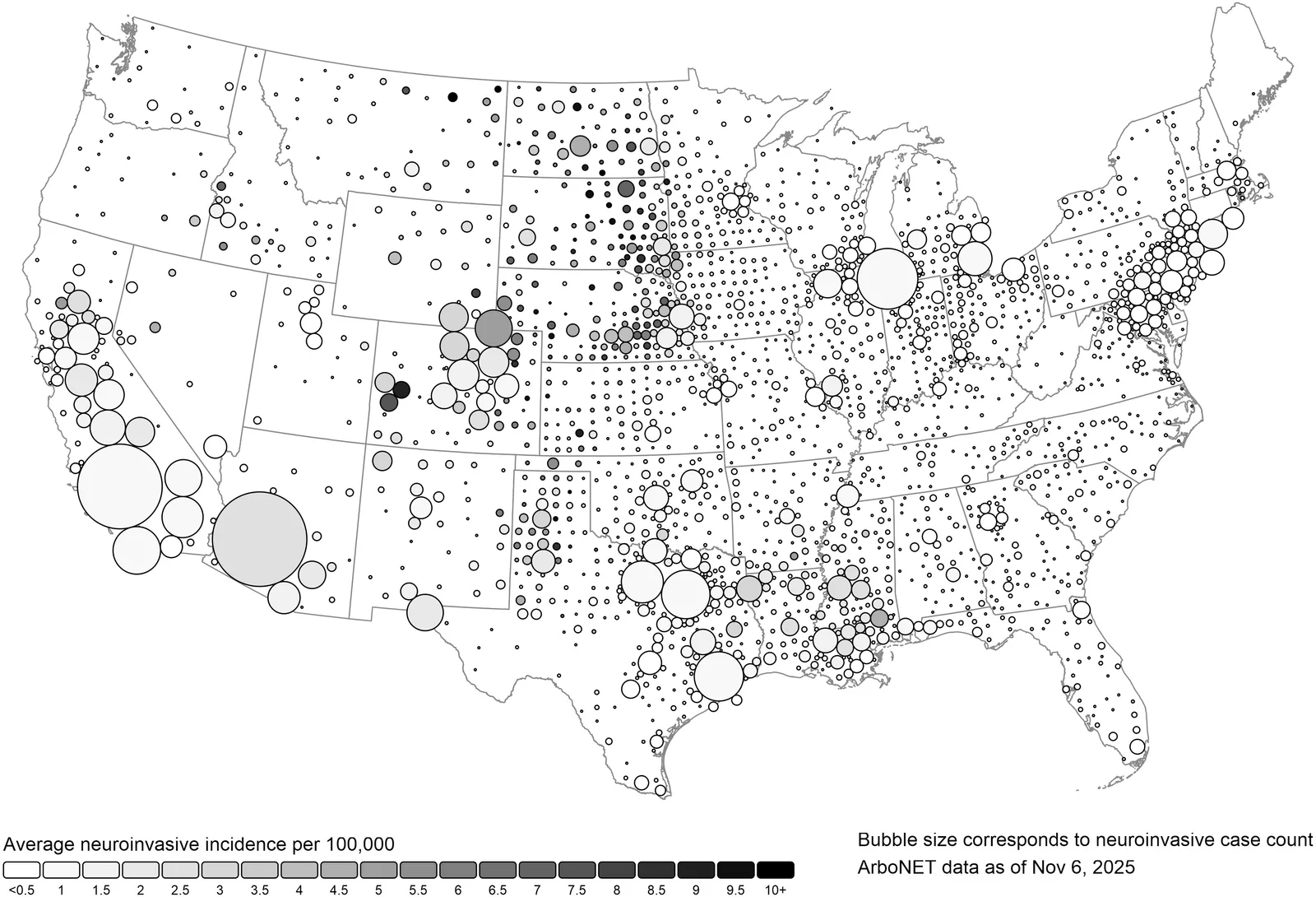
West Nile virus neuroinvasive disease case burden and incidence reported to the US CDC by U.S. county of residence, 1999–2024. The size of the circle corresponds to cumulative number of neuroinvasive disease cases by county of residence. Circles have been placed relative to the county but do not overlap so might not represent the exact geographic location. Cumulative incidence per 100,000 population is shown by the color shading with darker colors indicating high incidence.

Since its initial spread across the contiguous United States, WNV has caused a median 1,200 neuroinvasive disease cases and 120 deaths annually during the period 2005–2024 (Figure 1). However, during the last 10 years, the median was 1,300 disease cases and 130 deaths.^1^^,^^3^ It is possible that changing environmental conditions and human behaviors (e.g., irrigation practices) could influence WNV transmission and outbreaks. The cumulative incidence of neuroinvasive disease cases is highest in West North Central, Mountain, and South Central U.S. states (Figure 2).^3^^,^^6^ In contrast, the highest disease burden tends to be in areas with larger population centers, such as Chicago, IL; Houston, TX; Los Angeles, CA; or Phoenix, AZ.^6^ Canada has a lower burden of disease than the United States but has higher incidence in their Prairie Provinces across the border from West North Central states.^7^ Since 2018, Europe and other regions, including Israel and Tunisia,^8^^,^^9^ have experienced increasing WNV transmission and outbreaks.^1^^,^^10^

Despite the occurrence of thousands of disease cases annually in the United States, the location of these cases varies from one year to the next because of factors influencing circulation. These factors include changes in the bird and mosquito populations and immunity levels, presence of mosquito control and mosquito susceptibility to insecticides, weather patterns, population demographics, and human behaviors (e.g., increased awareness, testing, time spent outside).^11^ This variability of WNV incidence is best depicted on a finer geographic scale (Figure 1). For instance, the largest focal WNV outbreak occurred in Maricopa County, AZ, during 2021.^3^^,^^12^ A total of 1,476 disease cases, including 951 neuroinvasive disease cases, were reported that year in comparison with only 4 neuroinvasive cases during 2020. The same annual variation was observed in Dallas County during an outbreak in 2012.

### Future course of action.

#### Mosquito surveillance and control.

WNV prevention relies heavily on mosquito control programs. Integrated mosquito management (IMM) strategies include surveillance of mosquito species composition, abundance, and virus activity; larval habitat reduction; larviciding; and adulticiding via ultra-low-volume applications.^13^^,^^14^ Although IMM is the gold standard, mosquito control program capacities to implement it are inconsistent among jurisdictions. Many U.S. jurisdictions lack funding, structured mosquito surveillance, control capacity, or insecticide resistance monitoring, thus limiting the effectiveness of control efforts and inconsistent protection across regions.^15^^,^^16^ Reliance on a limited set of insecticides for public health use presents challenges because of widespread resistance in key WNV vectors, thus reducing insecticide effectiveness when resistance is not managed.^17^^–^^19^ Public concern regarding insecticide use further complicates ultra-low-volume operations despite data indicating a lack of evidence for human health risk when mosquito control products are applied according to label instructions.^20^^–^^22^ Targeted communication strategies emphasizing WNV disease risk and increasing awareness of the public health value of ultra-low-volume adulticides and larvicides are needed to support mosquito control activities.^23^

Evidence from multiple jurisdictions demonstrates that effective routine and emergency response depends on sustained mosquito surveillance and control infrastructure. In California, a long-standing, integrated surveillance system has enabled timely, targeted WNV interventions through close interagency collaboration and sustained baseline capacity.^24^ During the 2012 WNV epidemic in Texas, mosquito infection metrics and vector indices provided early indicators of transmission risk before peaks in human cases, allowing earlier escalation of control measures.^25^ Routine mosquito surveillance needs to be in place to establish baseline thresholds and interpret potential changes in risk in real time (Table 1). Without sustained surveillance and control infrastructure, response becomes reactive rather than preventive.

**Table 1:** Activities needed to improve existing and add additional WNV prevention and control measures^17^

Measure	Activity
PPBs	Advance understanding of and increase use of evidence-based public health communications tools to increase adherence to PPBs
	Develop and evaluate innovative strategies to increase adherence to PPBs, particularly at times of higher WNV transmission
Mosquito control	Collect and analyze existing mosquito surveillance and treatment datasets to identify conditions and interventions that reliably produce low WNV transmission and vector indices, so effective strategies can be replicated
	Advance field trials of mosquito population-suppression approaches (e.g., sterile insect technique, incompatible male releases, gene-based tools) tailored to *Culex* WNV vectors
	Develop and deploy next-generation chemical and biological control agents, along with innovative insecticide delivery platforms, to improve efficacy, overcome resistance, and achieve sustained suppression of *Culex* WNV vectors
	Evaluate existing ecologically targeted interventions that disrupt enzootic WNV amplification, including host-focused strategies
	Develop, validate, and deploy artificial intelligence-enabled mosquito identification tools to build entomological capacity across jurisdictions, enabling accurate species identification in resource-limited settings and strengthening surveillance and control decision-making
	Increase flexibility and coordination of regulatory processes for novel mosquito control technologies
Vaccine	Develop a TPP that outlines safety, immunogenicity, and efficacy criteria requirements
	Demonstrate immune correlates of protection in suitable animal models
	Conduct phased human clinical efficacy assessments facilitated with rapid diagnostic assay for efficacy determinations
	Foster early collaboration and engagement between manufacturers and the US Food and Drug Administration on key effectiveness endpoints and appropriate regulatory pathway, including potential expedited programs
	Conduct market forecast analysis to support commercial interest
	Encourage US Biomedical Advanced Research and Development Authority (BARDA) funding and establish potential for U.S. governmental subsidization and criteria metrics for such funding for regulatory market approval
	Develop diagnostic assay capable of distinguishing between natural WNV infection and prior vaccination (DIVA)
mAb used as preexposure prophylaxis and treatment of at-risk groups	Develop a TPP that outlines target population, safety, antibody durability, and efficacy criteria requirements
	Foster early collaboration and engagement between manufacturers and the US Food and Drug Administration on key effectiveness endpoints and appropriate regulatory pathway, including potential expedited programs
	Conduct phased human clinical studies and/or animal assessments (if applicable) to assess prophylactic and therapeutic mAb efficacy, using a rapid WNV diagnostic assay, and evaluate durability over a transmission season
	Conduct market forecast analysis to support commercial interest
Rapid WNV diagnosis	Develop a TPP that specifies assay sensitivity and specificity performance requirements aligned with their intended use in supporting vaccine and mAb efficacy assessments
	Conduct direct assessment of assay performance in applicable clinical samples
	Conduct market forecast analysis to support commercial interest

mAb = monoclonal antibody; PPB = personal protective behavior; TPP = target product profile; WNV = West Nile virus.

For mosquito control, sustained and increased investment in local IMM infrastructure is needed, and development and investment into stocks of materials and resources to control outbreaks. In addition, new chemical tools and novel technologies should be developed to complement existing control tools.^26^^,^^27^

#### Personal protective behaviors.

Personal protective behaviors (PPBs) aimed at reducing mosquito bites and subsequent WNV disease risk include using an Environmental Protection Agency-registered insect repellent, wearing permethrin-treated clothing, wearing long sleeves and pants when outdoors, and using window screens or air conditioning to keep mosquitoes outside, particularly during peak biting times.^28^ However, PPBs can lack effectiveness because of low adherence by the public (e.g., not avoiding peak biting times) and imperfect practice (e.g., suboptimal application of insect repellent).^29^^–^^32^ Public health authorities should ensure the population is aware of WNV disease risk with the use of potential risk meters (e.g., Skeeter Meter) given PPBs are more likely practiced when concern for WNV disease is higher.^29^ Additional partners, like the Vector-Borne Centers of Excellence should develop and assess innovative strategies that could lead to greater adherence to PPBs, and determine the effectiveness of PPBs in reducing WNV incidence (Table 1).

#### WNV vaccine.

Vaccines are used effectively to prevent WNV disease in horses and other animals, but no human WNV vaccines are currently licensed.^27^ However, an age- and incidence-based WNV vaccination program could prevent 37% of all neuroinvasive disease cases and 63% of WNV-related deaths if vaccines were provided to adults aged ≥60 years in states with an annual WNV neuroinvasive disease incidence of ≥0.5 per 100,000.^33^

Several vaccine candidates advanced through phase 1 human clinical trials and one advanced through phase 2 (Table 2).^34^ However, the development of WNV vaccines has not progressed because of the variability and unpredictability of WNV incidence and a lack of immune correlate of protection, both of which present substantial scientific and financial challenges to completing traditional phase 3 trials.^35^ A potential path forward for licensure of a human WNV vaccine could include one of the US Federal Drug Administration (FDA) expedited programs (i.e., Accelerated Approval, Fast Track) as has occurred for other vaccines (Table 1).^36^^,^^37^ Defining the market potential for a human WNV vaccine is also needed to drive financial investment and development by manufacturers.^35^

**Table 2:** WNV vaccine candidates tested in human clinical trials*

Vaccine Type, Name, and Trial	Age of Participants (years)	No. Participants Enrolled	No. and Timing of Doses	Immunogenicity	Serious Adverse Events Attributed to Vaccine	ClinicalTrials.gov Number
Live attenuated chimeric						
WN/DEN4-3′Δ30 (NIAID, NIH)						
Phase 1 trial, completed 2005	18–50	56	1 dose	55%–75% seroconversion (increase in PRNT_60_ by a factor of ≥4 at day 28 or 42^†^	None	NCT00094718
Phase 1 trial, completed 2009	18–50	26	2 doses, 6 months apart	89% seroconversion after second dose (increase in PRNT_60_ by a factor of ≥4 at day 28 or 42	None	NCT00537147
Phase 1 trial, completed 2016	50–65	28	2 doses, 6 months apart	95% seroconversion after first dose by day 90 (PRNT_50_ ≥1:10)	None	NCT02186626
ChimeriVax-WN02, YFV 17D backbone (Sanofi Pasteur, Lyon, France)						
Phase 2 trial, completed 2009						
Part 1	18–40	112	1 dose	>96% seroconversion (increase in PRNT_50_ by a factor of ≥4 at day 28)	None	NCT00442169
Part 2	≥41	96	1 dose	Approximately 96% seroconversion (increase in PRNT_50_ by a factor of ≥4 at day 28); GMT and viremia tended to be higher among persons ≥65 years of age	None	NCT00442169
Phase 2 trial, completed 2009	≥50	479	1 dose	92%–95% seroconversion (increase in PRNT_50_ by a factor of ≥4 at day 28)	None	NCT00746798
DNA						
VRC-WNVDNA017-00-VP, first generation (NIAID, NIH, DVBD, CDC)						
Phase 1 trial, completed 2008	18–50	15	3 doses, 4 weeks apart	100% had neutralizing antibodies detected (PRNT_50_) at 4 weeks and 24 weeks after third dose; titers were similar to those elicited in horses 3 weeks after one dose of WNV DNA vaccine	None	NCT00106769
VRC-WNVDNA020-00-VP, second generation (NIAID, NIH)						
Phase 1 trial, completed in 2007	18–65	30	3 doses, 4 weeks apart	>96% had neutralizing antibodies detected (EC_50_) at 4 weeks after third dose	None	NCT00300417
Recombinant subunit						
WN-80E (Hawaii Biotech; Honolulu, HI, USA)						
Phase 1 trial, completed 2009	18–45	25	3 doses, 4 weeks apart	Unpublished	Unpublished	NCT00707642
Inactivated whole virus						
HydroVax-001, hydrogen peroxide inactivated (Najit Technologies; Beaverton, OR, USA)						
Phase 1 trial, completed 2016	18–49	51	2 doses (1 *µ*g or 4 *µ*g), 4 weeks apart	31%–50% seroconversion at 15 days after second dose (PRNT_50_ ≥20 with or without complement enhancement)	None	NCT02337868
Formalin inactivated (Nanotherapeutics; Alachua, FL, USA)						
Phase 1–2 trial, completion date unknown	≥18	320	2 doses, 3 weeks apart; booster on day 180	All doses elicited immune response, with peak antibody responses after booster dose (microneutralization)	None	None

CDC = US Centers for Disease Prevention and Control; EC = effective concentration; GMT = geometric mean titer; NIAID = National Institute of Allergy and Infectious Diseases; NIH = National Institutes of Health; PRNT = plaque reduction neutralization test; WNV = West Nile virus.

* Adapted from Gould et al.^34^

^†^ PRNT titers expressed as the reciprocal of the last serum dilution demonstrating the given reduction in plaque counts (50% or 60%).

#### WNV monoclonal antibodies.

Similar to other recently licensed monoclonal antibodies aimed at preventing disease,^38^ anti-WNV monoclonal antibodies (mAbs) hold promise as seasonal preexposure prophylaxis in people at higher risk of severe WNV disease and death.^39^ In addition, mAbs could be potentially used to treat these same individuals who develop disease. Because mAbs provide passive immunity, these products can be given to people who cannot develop an immune response to a vaccine, including older patients with multiple comorbidities or immunocompromised patients, who can have up to a 250-times higher risk for severe WNV disease (Table 3).^40^^,^^41^ In 2021, 6.6% of U.S. adults self-reported being immunosuppressed because of health conditions, prescriptions, or medical treatments, which represents approximately 17.8 million adults in the United States.^42^

**Table 3:** Risk factors for severe WNV disease or WNV-related death

Characteristic	Risk Factors
Demographics	Older age^47^; incidence increasing with each decade of life^47^^,^^48^
	Male sex,^47^^,^^48^ particularly among those ≥50 years of age
Clinical features	Chronic kidney disease
	History of cancer
	History of alcohol abuse
	Diabetes
	Hypertension
	Immune suppression, including use of B-cell depleting monoclonal antibodies^40^^,^^41^^,^^49^^,^^50^
Other	Infection acquired through solid organ transplantation^1^^,^^51^
	Anti-interferon antibodies^52^^,^^53^
	Polymorphisms in genes involved in antiviral activity (e.g., CCR5, IRF3, MX1)^54^^,^^55^

WNV = West Nile virus.

Currently, limited data are available regarding use of mAbs to treat WNV disease in humans and none on their use as a potential prophylactic agent. A phase 1 study of a recombinant humanized version of mAbs targeting the E protein of WNV in healthy adults found the product to be well-tolerated and had a half-life of 27 days.^43^ However, a phase 2 clinical trial exploring the use of the product to treat neuroinvasive disease was never completed because of low enrollment.^44^^,^^45^

Work is needed to better define the potential scope of people who could benefit from receiving mAbs, define characteristics of these products (e.g., side effects, duration of protection), assess the cost-effectiveness of this strategy, determine applicability of expedited regulatory review, and define the market potential (Table 1).

#### WNV rapid diagnostic testing.

Testing for WNV is currently obtained through commercial laboratories or state public health laboratories with a typical turnaround time of 1 to 8 days. Having a WNV diagnostic test that provides results in <4 hours and readily available in clinics or hospital settings (i.e., rapid diagnostic) could improve clinical care by reducing unnecessary diagnostic testing and potentially harmful and costly treatments (e.g., antimicrobial use). It would also overcome one of the current obstacles for timely antiviral or antibody treatment and support phase 3 clinical efficacy trials for a WNV vaccine and mAbs by allowing for rapid identification of WNV-infected patients.^1^

Recent work indicating increased sensitivity and prolonged viremia in whole blood could be a target for rapid diagnostic test development and help with making a timely diagnosis, particularly for immunocompromised patients, in whom viremia can be prolonged and antibody development delayed.^40^^,^^46^

The CDC is collaborating with government, academic, and industry partners to develop target product profiles defining minimal and preferred characteristics for a WNV vaccine, prophylactic mAb, and a rapid WNV diagnostic test to facilitate future development. The CDC also anticipates funding market forecast analyses to determine market potential.

## CONCLUSION

Mosquito control and PPBs are the only measures currently available for WNV disease prevention. In the United States, the median number of severe WNV cases and deaths occurring annually have not decreased despite these measures. Improving existing mosquito surveillance and control and measures aimed at increasing uptake of PPBs, and developing and implementing additional measures, such as human WNV vaccines, anti-WNV mAbs used as seasonal preexposure prophylaxis or for treatment in at-risk populations, and more rapidly diagnosing WNV disease, are needed to reduce WNV morbidity and mortality. Further collaboration within and outside of the federal government is needed to ensure optimal use and availability of current and additional lifesaving measures.
